# Floral Color Diversity: How Are Signals Shaped by Elevational Gradient on the Tropical–Subtropical Mountainous Island of Taiwan?

**DOI:** 10.3389/fpls.2020.582784

**Published:** 2020-12-17

**Authors:** King-Chun Tai, Mani Shrestha, Adrian G. Dyer, En-Cheng Yang, Chun-Neng Wang

**Affiliations:** ^1^Department of Life Science, National Taiwan University, Taipei, Taiwan; ^2^Institute of Ecology and Evolutionary Biology, National Taiwan University, Taipei, Taiwan; ^3^School of Media and Communication, RMIT University, Melbourne, VIC, Australia; ^4^Department of Entomology, National Taiwan University, Taipei, Taiwan

**Keywords:** flowers, bee vision, phylogeny, community, altitude, tropical–subtropical, island

## Abstract

Pollinators with different vision are a key driver of flower coloration. Islands provide important insights into evolutionary processes, and previous work suggests islands may have restricted flower colors. Due to both species richness with high endemism in tropical–subtropical environments, and potentially changing pollinator distributions with altitude, we evaluated flower color diversity across the mountainous island of Taiwan in a comparative framework to understand the cause of color diversity. We sampled flower color signaling on the tropical–subtropical island of Taiwan considering altitudes from sea level to 3300 m to inform how over-dispersion, random processes or clustering may influence flower signaling. We employed a model of bee color space to plot loci from 727 species to enable direct comparisons to data sets from continental studies representing Northern and Southern Hemispheres, and also a continental mountain region. We observed that flower color diversity was similar to flowers that exist in mainland continental studies, and also showed evidence that flowers predominantly had evolved color signals that closely matched bee color preferences. At high altitudes floras tend to be phylogenetically clustered rather than over-dispersed, and their floral colors exhibited weak phylogenetic signal which is consistent with character displacement that facilitated the co-existence of related species. Overall flower color signaling on a tropical–subtropical island is mainly influenced by color preferences of key bee pollinators, a pattern consistent with continental studies.

## Introduction

Floral color is a key functional trait that affects plant–pollinator interactions (Oberrath and Böhning Gaese, 1999; Bradshaw and Schemske, 2003; Ômura and Honda, 2005), contributing to biodiversity and structure of plant communities (Sargent and Ackerly, 2008; Schemske et al., 2009). The variation of floral color diversity (FCD) may reflect how plant–pollinator interactions and evolutionary history shape color signaling (Weevers, 1952; Chittka and Menzel, 1992; McEwen and Vamosi, 2010). Whilst there has been increased interest on the studies of FCD for continental floras around the world (Arnold et al., 2009a,b; Dyer et al., 2012; Shrestha et al., 2014; Gray et al., 2018), relevant studies of island floras are rarely reported (Makino and Yokoyama, 2015; Shrestha et al., 2016).

Island data can provide important insights for a better understanding the global patterns of floral color resulting from plant–pollinator interactions, and how threats like species invasion, habitats loss and/or pollinator extinction (Cox and Elmqvist, 2000; Brooks et al., 2002; van Kleunen et al., 2015) may impact environments. Compared to continental environments, islands can provide an assessable model with insular nature to study the FCD (Emerson and Gillespie, 2008). Interestingly, previous studies report that oceanic island floras [e.g., Macquarie Island (MI) (Shrestha et al., 2016), Juan Fernández Islands (JFI) (Bernardello et al., 2001) and sub-Antarctic Campbell Island (Lord et al., 2013) display simple and inconspicuous colors and have low FCD. Even on larger oceanic islands, floras in New Zealand (NZ) mountains consist of constrained flower color signals, exhibiting much lower FCD (Bischoff et al., 2013b) when compared to its adjacent continental island of Australia (Webb and Kelly, 1993; Dyer et al., 2012). It’s worth noting that the low species richness of these oceanic islands, e.g., MI (37 angiosperm species, de Salas and Baker (2015) and JFI (152 species), may more or less determine their low FCD, as plant communities with larger species richness would often, but not always, have greater FCD (Arnold et al., 2009b; Shrestha et al., 2014; Bergamo et al., 2018; Gray et al., 2018).

Oceanic island plants were mainly colonized from continents within a short evolutionary history and often lacks guilds of pollinators, thus low FCD was expected. Unlike oceanic islands, Taiwan is an atypical island, i.e., isolation with partial reconnection during glaciation and it has been repeatedly in contact with the Asian continent (Voris, 2000), which allows plants to readily migrate into Taiwan (Hsieh, 2002; Huang, 2011; Li et al., 2013). Moreover, Taiwan is situated on the transition zone between subtropical and tropical region, potentially contributing to a greater FCD, as biota have long been thought to exhibit greater color diversity in tropics than high latitudes (Wilson and von Neumann, 1972; Adams et al., 2014). The islands of Taiwan are located at the boundary between the Holarctic and Paleotropical floristic kingdoms indicating the diverse evolutionary origins of local flora (Hsieh, 2002). Indeed, Taiwan harbors greater species richness (up to 3,500 flowering species) and higher specific endemism (26.1% of native plants) than other oceanic islands (Hsieh, 2002), we thus might expect a greater FCD in Taiwan, especially with respect to other island studies.

In addition to having high species richness, Taiwan also has mountainous topography, with steep altitudinal gradients up to 3952 m a.s.l. and accessible flowering plants to 3300 m a.s.l. within a relatively short distance. This creates altitudinal vegetation zones from tropical/foothill lowland evergreen broad-leaved forest to subtropical evergreen and temperature deciduous mountain forest, and to mountain peaks as cool temperate upper-montane coniferous and subalpine boreal forest or alpine tundra (Li et al., 2013). The percentage of flowering plant endemism in Taiwan therefore increases dramatically along altitude from 15% at sea level to reach 60% among most mountain peaks (Hsieh, 2002). This provides us with an appealing opportunity to incorporate how floral colors are shaped along altitudinal gradients of the tropical–subtropical island, as previous works have studied the variation of FCD along altitudes in several continents (Arnold et al., 2009b; Shrestha et al., 2014; Gray et al., 2018), but to the best of our knowledge this has not been tested on an island.

Considering mountainous environments, Bergamo *et al.* analyzed 71 plant species in 700–1800 m a.s.l. and found the FCD was greater in low altitudes than that in middle and high altitudes (Bergamo et al., 2018), whereas Shrestha et al. (2014) analyzed 107 plant species in Nepal and found a converse pattern, i.e., greater the FCD in subalpine region (3000–4100 m, *n* = 61) than subtropical regions (900–2000 m, *n* = 46). Interestingly, in the Himalayan mountains of Nepal there is also a high diversity of bumblebees and some other insects (Thapa, 2000; Williams et al., 2010) that act as pollinators of flowering plants (Shrestha et al., 2014; Paudel et al., 2015, 2016, 2018). These studies suggested that the species richness (represented by sample sizes) may be a main factor influencing FCD, and altitude as a factor may only mediate probability of species richness depending upon temperature range in a particular region of the world. To contribute to our understanding of the complexity of FCD and climatic conditions due to changing altitude, our work included a large sample size in each altitude group (>140 species) and covered an entire altitude gradient over which flowering plants exist in Taiwan (from 0 to 3300 m). This enables formally testing if species richness is a key factor to determine the FCD in an environment.

In addition, floral color assembly (FCA) demonstrates the similarity of floral colors between species within a given region, i.e., if sympatric floras tend to display similar colors (cluster) or divergent colors (over-dispersion). When compared to FCD, an investigation of FCA can enable interpretations of how species evolutionary history and plant–pollinator interaction shape the floral colors within different plant communities (Gumbert et al., 1999; McEwen and Vamosi, 2010; Makino and Yokoyama, 2015).

Several studies used null models based on random assembly to evaluate the pattern of FCA, and revealed that FCA was non-random in most plant communities and highly shaped by the plant–pollinator interaction (de Jager et al., 2011; Muchhala et al., 2014; Makino and Yokoyama, 2015; Ohashi et al., 2015; Kemp et al., 2019), although evidence of random assembly also exists for the continental island of Australia (Shrestha et al., 2019b). Competition for pollinator visitations was the major selection force to shape the FCA of plant communities in high latitudes and high altitudes, which often caused floral color over-dispersion (Muchhala et al., 2014; van der Kooi et al., 2016; Bergamo et al., 2018), but there is also some evidence of clustering at high altitudes (Bergamo et al., 2018). As both number and abundance of pollinators are thought to generally decrease with increasing altitudes (Warren et al., 1988; Bingham and Orthner, 1998), but see Shrestha et al. (2014) for a different effect in the Himalayan mountains, it is plausible this may affect FCA.

At a global scale large islands have relatively more bee species (ca. 0.0011–0.0420 spp. per square kilometer, and Taiwan 0.005) than are present on continental land masses (ca. 0.0004–0.0015) (Buchmann, 1994). Bees are often more effective in pollination than other insects, e.g., bees deliver more pollen in each visitation, and visit more flowers per minute than the flies (Bingham and Orthner, 1998; Bischoff et al., 2013a) as well as often exhibiting flower constancy (Chittka et al., 1999). In our current study, we thus not only investigated the floral colors in the human color category, but also translated their reflectance spectra into color loci in sensory color space of the bee (bee hexagon) which permits a robust interpretation of how important bee pollinators perceive floral color signals (Chittka, 1992; Dyer, 2006; Kemp et al., 2019; Shrestha et al., 2019b). This advanced technique is possible because for bees there is detailed knowledge about their phylogenetically conserved trichromatic vision including receptors, neural processing and calibration with behavioral data (Chittka, 1992; Dyer et al., 2011; Garcia et al., 2017; Shrestha et al., 2019a,b). The bee color hexagon model was used to quantify the FCD (Shrestha et al., 2014, 2016), and was also used to evaluate the FCA by applying the mean pair-wise distance (MPD) of the color loci (McEwen and Vamosi, 2010; Makino and Yokoyama, 2015; Shrestha et al., 2019b).

Studies of floras from both Southern and Northern Hemispheres consistently exhibited a predominant direction of color loci in bee hexagon (Chittka et al., 1994; Dyer et al., 2012; Shrestha et al., 2014, 2019b), which overlapped with the short-wavelength “blue” color preferences of several bee species that have been formally tested (Giurfa et al., 1995; Raine and Chittka, 2005; Dyer et al., 2016). Such distinct color preferences likely exist due to the overlap of the phylogenetically conserved color photoreceptors in bees from around the world (Peitsch et al., 1992; Giurfa et al., 1995; Raine and Chittka, 2005; Morawetz et al., 2013; Dyer et al., 2016), and strongly suggest that the bee color preferences are the major selection force that shape the floral colors in regions where bees are common pollinators (Shrestha et al., 2019b). Indeed, when bees are not the pollinator of flowers then very different distributions of flower colors are observed (Lunau et al., 2011; Shrestha et al., 2013, 2016, 2019a; Burd et al., 2014; Camargo et al., 2019).

The multiple origins of Taiwan flora and complex island habitats thus may generate unique floral colors as flowers adapt to different pollinators along elevation gradients. We therefore would like to test whether flower color diversity is comparable to these adjacent continent and islands. We also would like to ask whether flower color is constrained for attracting limited pollinators on islands or diverges for competing pollinator visiting. Our null position is that flower color in Taiwan is not significantly different to mainland continental studies. Thus, in the current study, we firstly evaluate whether the floras in tropical–subtropical island Taiwan exhibited low FCD consistent with other island studies, or if the favorable conditions of a tropically located mountain island may promote higher FCD consistent with, or even greater than, mainland continental studies. We secondly investigate if there is evidence of variation of FCD considering three defined different altitudinal zones in TW, and evaluate if any observed variation might be dependent upon species richness. For our analyses we consider the evidence or whether FCA of plants in TW are over-dispersed, random or clustered based on low, mid and high-altitude plants using the phylogenetic-informed analysis, whereby we could interpret the possible driving force and ecological process accounting for the patterns of FCA. Finally, we use a comparative framework to evaluate if bee color preferences are also a likely factor influencing the distribution of floral colors in Taiwan, as has been recently demonstrated in other parts of the world.

## Materials and Methods

### Study Area and Sample Collection

**FIGURE 1:**
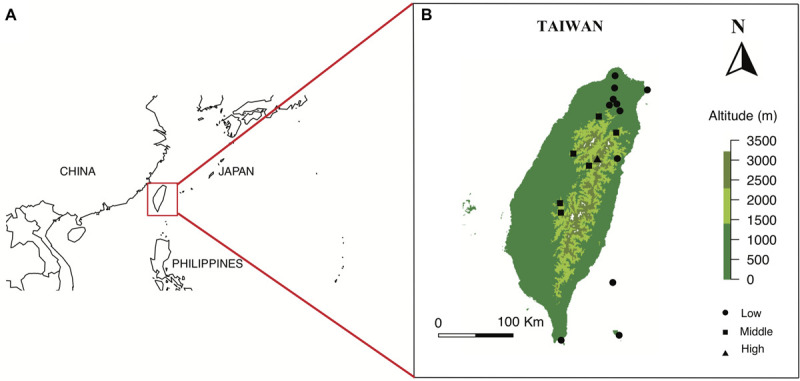
Location of Taiwan in red square **(A)**, and the maps of 18 sampling sites from three altitudinal regions in Taiwan **(B)**. Maps are prepared using package Maps in R version 3.4.4.

The main island of Taiwan lies in East Asia located in between 21.916107 N - 25.086991 N, 120.787456 E - 121.899446 E with a land area of 35,808 square kilometers. The island is surrounded by South and East China Seas, and at the nearest point is 130 km from any other major continental land mass (Figure 1). The main island lies in a complex tectonic region of Yangtze Plate to the west and north, the Okinawa Plate on the north-east, and the Philippine Mobile Belt on the east and south, formed approximately four to five million years ago (Wu, 1978; Biq et al., 1985). Moreover, many high mountains in Taiwan (over 100 peaks over 3000 m a.s.l.) have created topographically isolated habitats and fast-changing climatic zones (from tropical low land forest to alpine tundra) along elevation changes. The multiple origins of Taiwan flora and complex island habitats thus may have potentially generated very unique FCD in different habitats along elevation. However, very little was known about Taiwanese or even Asian flower coloration with respect to animal pollinator color vision prior to our study.

**FIGURE 2:**
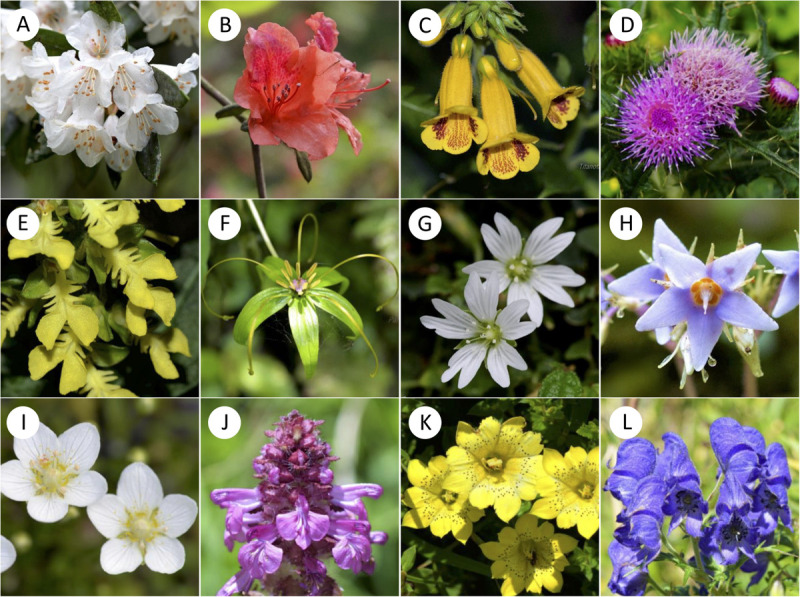
Examples of representative native flowering species from low **(A–D)**, middle **(E–H)** and high **(I–L)** altitudes of Taiwan. **(A)** *Rhododendron formosanum*; **(B)** *Rhododendron oldhamii*; **(C)** *Titanotrichum oldhamii*; **(D)** *Cirsium japonicum* var. *australe*; **(E)** *Odontochilus bisaccatus*; **(F)** *Paris polyphylla* var. *stenophylla*; **(G)** *Stellaria arisanensis*; **(H)** *Conandron ramondioides*; **(I)** *Parnassia palustris*; **(J)** *Pedicularis verticillata*; **(K)** *Gentiana scabrida* var. *punctulata*; **(L)** *Aconitum fukutomei*.

We selected our study sites in the Taiwanese main island and two nearby offshore islands named Green Island and Orchid Island. We classified these sites into different altitudinal regions, i.e., low (0–900 m a.s.l.), middle (1500–2200 m a.s.l.) and high-altitude regions (2800–3300 m a.s.l.) (Supplementary Table S1). Sample sites in low-altitude comprise multiple types of vegetation zones including coastal, tropical and subtropical mountain zonal forest; middle sites are dominated by subtropical evergreen cloud forest, while the high-altitude sample site contained high-mountain coniferous woodlands and forests (Li et al., 2013). We chose a total of 18 sample sites from three altitudinal regions: 11 sites in low, 6 sites in middle, and 1 site in high-altitude (details in Supplementary Table S1). These sampling sites cover major altitudinal vegetation zones in Taiwan. We mostly choose the National Parks in different altitudinal regions, which were well-studied and represent all vegetation types of the study region. The number of sites tested reflected availability in respective National Parks. We sampled one high-altitudinal site covering a 10 km^2^ mountain range of several higher than 3200 m mountain peaks at the Taroko national park. All flower samples were native to Taiwan and were collected from March 2016 to September 2017, covering two periods of the peak blooming (Tai, 2018). Our dataset comprised of a total of 727 native flowering species (399 species in low-altitude, 186 species in middle-altitude, and 142 species in high-altitude) [see details Supplementary Table S2: species list and altitude, *Dryad digital repository* (Tai et al., 2020)] including ca 33% endemic species to Taiwan. Some example flowers collected at different altitudinal regions is given in Figure 2. We also used the data from Australia (Burd et al., 2014), Nepal (Shrestha et al., 2014), and Japan (Makino and Yokoyama, 2015) to enable a comparative framework to other regions.

### Floral Color Measurement

Floral colors were measured using an Ocean Optics spectrophotometer (Ocean Optics Inc., USB-4000+, United States) with a UV-VIS-NIR light source (Ocean Optics Inc., DH-2000-BAL, United States) and a quartz fiber-optic probe (Ocean Optics Inc., Lab-grade Reflection Probes, United States). The reflectance spectra were measured from 300 to 700 nm (see Supplementary Method S1) and then processed by the software OCEAN VIEW (Ocean Optics Inc., United States). Additional details for the measurement of reflectance spectra were given in Supplementary Method S1 and Supplementary Figure S1 for the details of marker point comparison to show the evidence of bees are the primary pollinator of flowering plants at low, middle, and high altitudes in Taiwan.

### Hymenopteran Color Space Modeling and Descriptors of Floral Colors

To analyze the FCD as perceived by bees, we used the bee color hexagon model which is widely accepted in relevant studies (Chittka, 1992; Chittka and Menzel, 1992; Dyer et al., 2012; Shrestha et al., 2014, 2019b; Kantsa et al., 2018; Kemp et al., 2019). In this model, the reflectance spectrum of each species is translated into a locus in a two-dimensional plane of the hexagon color space (Chittka, 1992) by employing photoreceptor sensitivities for a bee (350, 440, and 540 nm) (Dyer, 1999), standard D65 illumination (Judd et al., 1964) corrected for photon flux and assuming the visual system was adapted to the leaf-green background (reflectance spectra of the leaves for all samples in our study, see Supplementary Method S1). We categorized hexagon color space in to six sub-sectors (*BLUE*: B; *BLUE GREEN*: BG; *GREEN*: G; *UV-GREEN*: UG; *ULTRAVIOLET*: UV; *UV-BLUE*: UB) following the method used in Chittka et al. (1994). Each color locus in bee hexagon was also specified as Polar co-ordinate (a certain vector angle θ and vector magnitude *r*) or Cartesian co-ordinate (a certain *x* and *y*) (Chittka et al., 1994).

To quantitatively compare the overall FCD among different geographic regions and different altitudinal regions, we calculated the area of the minimum convex polygon (MCP) which encapsulate all color loci using the function ‘Polygon’ in R package SP (version no. 3.4.4).

For descriptors of floral spectral signals considering bee color vision, color hue can be defined as the vector angle θ of color loci in bee hexagon, while color contrast can be defined as the vector magnitude *r* from the achromatic center of color space that represents background information to the locus of a color (Chittka, 1992). Color distance is the Euclidean distance (Cartesian co-ordinate) between two color loci in bee hexagon (Chittka, 1992; Garcia et al., 2017).

### Phylogenetic Tree

**FIGURE 3:**
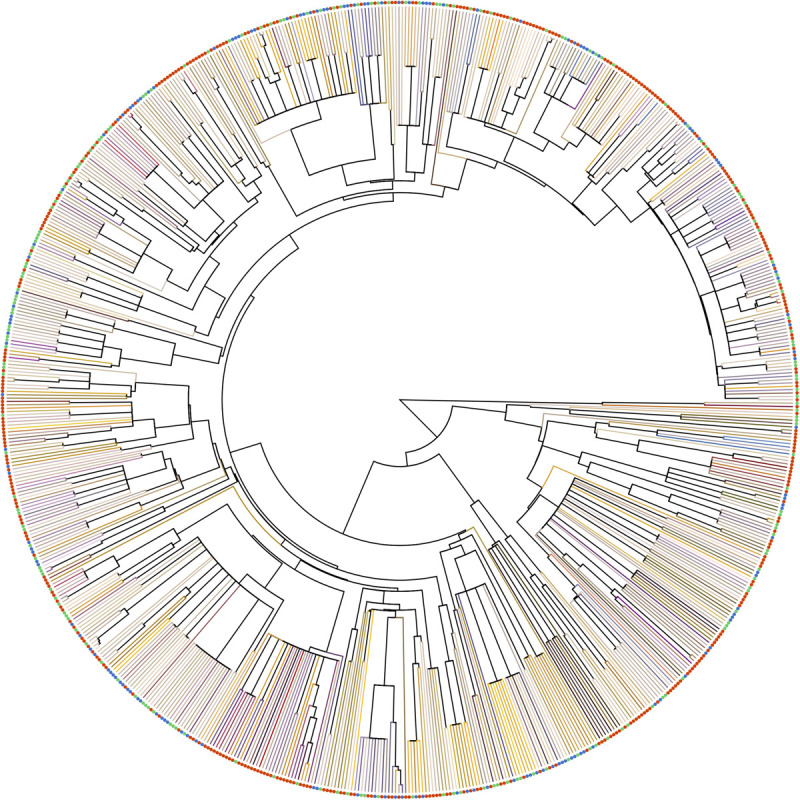
Phylogenetic relationship of the species in our samples. Terminal branches were painted as the floral color of each species in human vision, which was generated from the reflectance spectrum with function ‘spec2rgb’ of R package PAVO. Solid circles at the tip represents the altitude region of the species (red: low-altitude, green: middle-altitude, blue: high-altitude).

To access phylogenetic-informed analysis for our three altitudinal group data, we constructed the phylogenetic tree for all species using Phylomatic 3.0 (Webb and Donoghue, 2005), which was widely used in the multispecies analysis (Stournaras et al., 2013; Shrestha et al., 2014; Zanne et al., 2014; Ohashi et al., 2015). We used Wikström et al. (2001, 2015) to calibrate the many of the nodes and used other published references whereas our genus label phylogeny were based on Soltis et al. (2011) (see details in Supplementary Table S3). In the output phylogenetic tree, relationship among all family level and some genera level clades (e.g., in Rosaceae and Fabaceae) were left as unresolved polytomies (Figure 3). A phylogenetic distance of each pairwise species was computed with function ‘cophenetic.phylo’ in R package APE. The output tree Appendix A available in *Dryad Digital Repository* (Tai et al., 2020).

### Floral Color Assembly (FCA)

We used mean-pairwise distance (MPD) for the color loci in bee hexagon, which we termed MPD_color_, to evaluate the pattern of FCA in low, middle and high altitudes. First, we calculated the actual mean pairwise distance (actual MPD_color_) for all color loci of each altitude. This actual MPD_color_ will be compared with the mean of nulls MPD_color_ (indicating random assembly), which is generated from randomly resampling the color loci from species pool for 1,000 times. Species pool comprises all samples in our study and the sample size for each resampling is identical to the species number within that region.

The pattern of FCA is determined by the comparison between actual MPD_color_ and the mean of nulls MPD_color_. We computed all MPD_color_ and its significance *p*-value with function ‘ses.mpd’ in R package PICANTE (Kembel et al., 2010) (details provided in Supplementary Method S2).

### Phylogenetic Structure

We used mean-pairwise distance (MPD) to evaluate the pattern of phylogenetic structure of species in low, middle and high altitudes, except that phylogenetic distance rather than color distance is the variable analyzed here (thus termed MPD_phylo_). The pattern of phylogenetic structure was determined by the same procedure of comparison between actual MPD_phylo_ and the mean of nulls MPD_phylo_ as that of FCA described above. That the actual MPD_phylo_ was lower than the mean of nulls MPD infers species in that region tend to be closely related to each other (phylogenetic cluster). On the contrary, if actual MPD_phylo_ was larger than the mean of nulls MPD_phylo_ this would infer that species tend to be distantly related to each other (phylogenetic over-dispersion). Likewise, higher *p*-value indicates stronger phylogenetic over-dispersion, while lower *p*-value indicates phylogenetic cluster. We used the PICANTE function ses.mpd for these calculations (Kembel et al., 2010). In addition, we also calculated mean nearest taxon distance (MNTD_phylo_) to evaluate the phylogenetic structure and details for these metrics are given in the Supplementary Method S2.

### Phylogenetic Signal

We examined phylogenetic signal with the commonly used Blomberg’s K in previous studies (Blomberg et al., 2003; Muchhala et al., 2014; Shrestha et al., 2014). We calculated the Blomberg’s *K* values of the two key floral color descriptors: color hue (Shrestha et al., 2014), and color contrast (referred to as saturation in some studies) in bee hexagon (Lunau, 1990; Kantsa et al., 2017) of low, mid and high-altitude floras in our study using the function ‘phylosig’ in R package PHYTOOLS (Revell, 2012). We also performed Mantel test to evaluate the phylogenetic signal (Muchhala et al., 2014; Shrestha et al., 2014; Bergamo et al., 2018). Additional details of the calculations for Blomberg’s *K* and Mantel test are given in the Supplementary Method S3.

## Results

**FIGURE 4:**
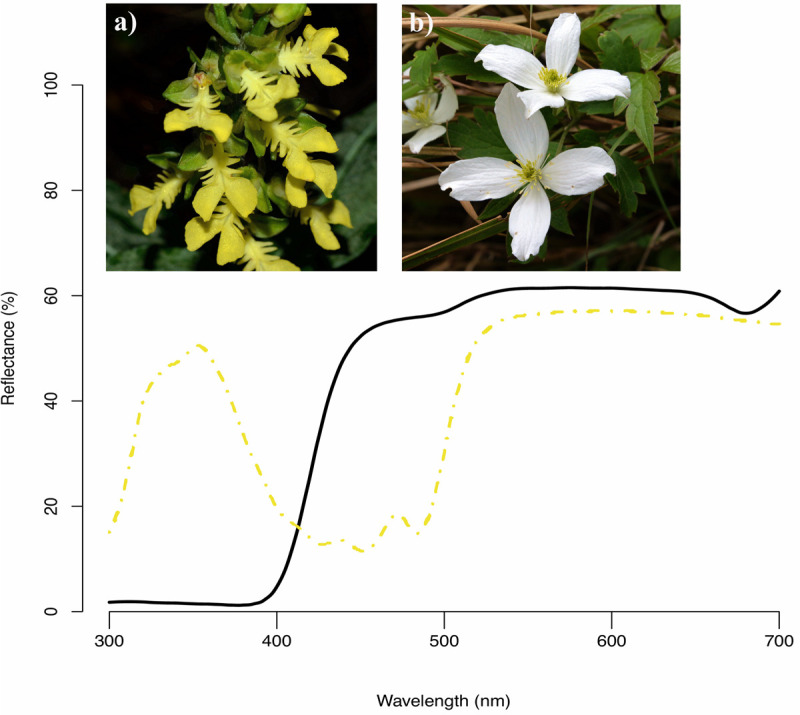
Example reflectance spectra of two native species in our study **(a)** *Odontochilus bisaccatus* (dash yellow curve, human yellow) and **(b)** *Clematis montana* (black line curve, human white).

Among 727 flowering plant species, the most common floral color in human vision was white (45%), followed by yellow (18%), pink (13%), and purple (11%) whereas species with green (4%), blue (3%), red (3%), and other/orange (3%) flowers were less frequent in Taiwan. Our analysis of their reflectance spectra showed that almost all white flowers are UV-absorbed white (e.g., Figure 4b), consistent with previous studies (Inouye and Pyke, 1988; Dyer, 1996; Kevan et al., 1996). Spectra of yellow flowers could be categorized into two types (Dyer, 1996) based on the presence of UV (67%, e.g., Figure 4a) and absence UV (33%) reflection.

### Floral Color Diversity (FCD)

**FIGURE 5:**
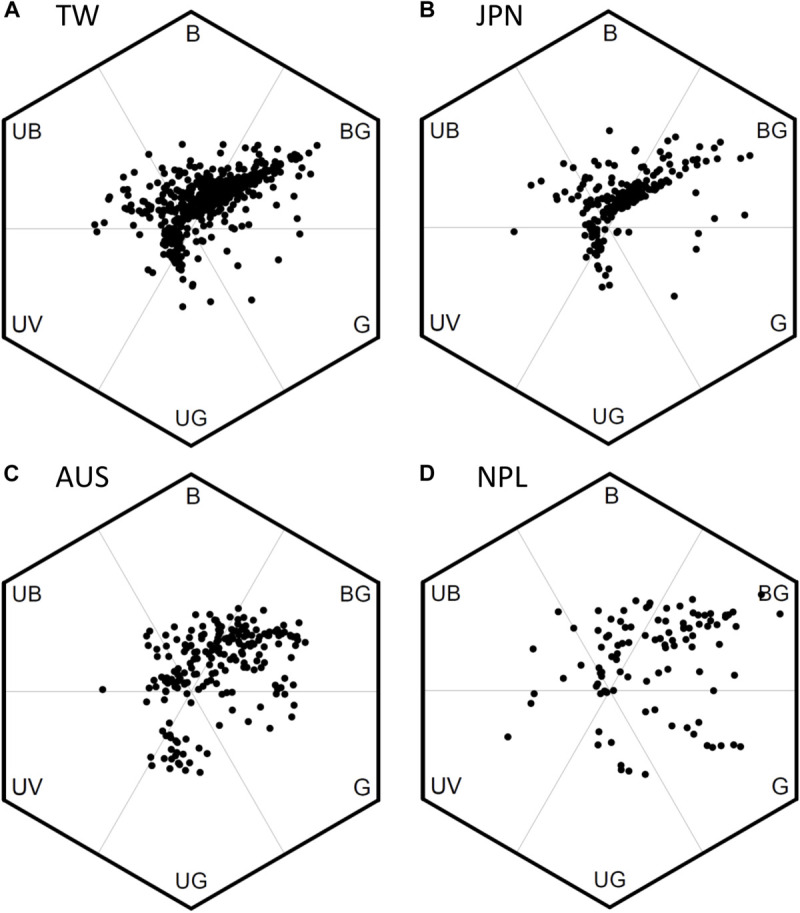
Bee color hexagon showing the floral color loci of species from four separate geographic regions. **(A)** 727 species of Taiwan; **(B)** 212 species of Japan (Makino and Yokoyama, 2015); **(C)** 201 species of Australia (Dyer et al., 2012; Burd et al., 2014); **(D)** 107 species of Nepal (Shrestha et al., 2014). We recalculated the color loci in bee hexagon of Japanese samples based on the published reflectance spectra in Makino and Yokoyama (2015).

We used bee color space to evaluate the pattern of FCD in Taiwan (TW) and compared the patterns to that of the continental island of Australia (AUS), the mountains of Nepal (NPL) and to Japan (JPN) (temperate continental islands) (Figures 5A–D). Color loci of floras in TW covered a broad range in bee hexagon (Figure 5A), which was comparable to that of continental floras from AUS (Figure 5C) and mountains of NPL (Figure 5D). The Area of MCP (representing FCD) of color loci in TW was 0.558 (hexagon squared units) which was larger than that (0.498) for AUS, but lower than that (0.783) for both NPL and JPN 0.588 (details Supplementary Table S5), thus suggesting overall a somewhat similar pattern to what has been previously observed.

The color loci of floras in TW concentrated to two distinct clusters in bee hexagon. One cluster has the greatest frequency toward the bee-BG vertex of bee hexagon, which can be termed ‘White Arm’ because most floral colors inside were human white (Figure 5A). Similarly, the other cluster lay toward bee-UG vertex can be termed ‘Yellow Arm’ as it was made up of human yellow flowers (Figure 5A). We also found similar ‘White and Yellow Arms’ in the pattern of JPN (Figure 5B), and these colors are also frequent flower hues from AUS (Figure 5C) and the mountains of NPL (Figure 5D), although the “arm” is less distinct.

**FIGURE 6:**
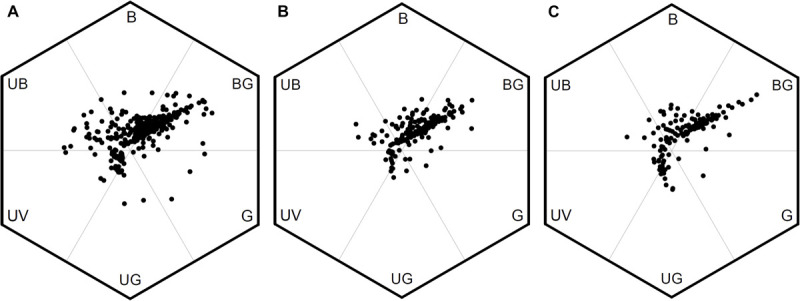
Bee color hexagon showing floral color loci of species from low, middle, and high altitudes in Taiwan. **(A)** 399 species from low-altitude, **(B)** 186 species from middle-altitude, and **(C)** 142 species from high-altitude.

We evaluated the variation of FCD between low, middle, and high altitudes in Taiwan by comparing the area of MCP of the color loci in bee hexagon (Figure 6). We found the area of MCP of low-altitude (0.546) was larger than that in high-altitude (0.304) and almost twice as that of middle-altitude (0.270). It was possible that the unequal sample size between different altitudes (from low to high: *n* = 399, 186 vs. 142) may bias the areas of MCP. We thus conducted permutation test to resolve this argument, and the result revealed that the areas of MCP of middle and high altitudes persisted to be lower than that of low-altitude (Supplementary Method S4 and Supplementary Figure S2). Thus, this result suggested that floras of low-altitude in Taiwan exhibited greater FCD than that of middle and high altitudes.

### Color Hexagon Sector

**FIGURE 7:**
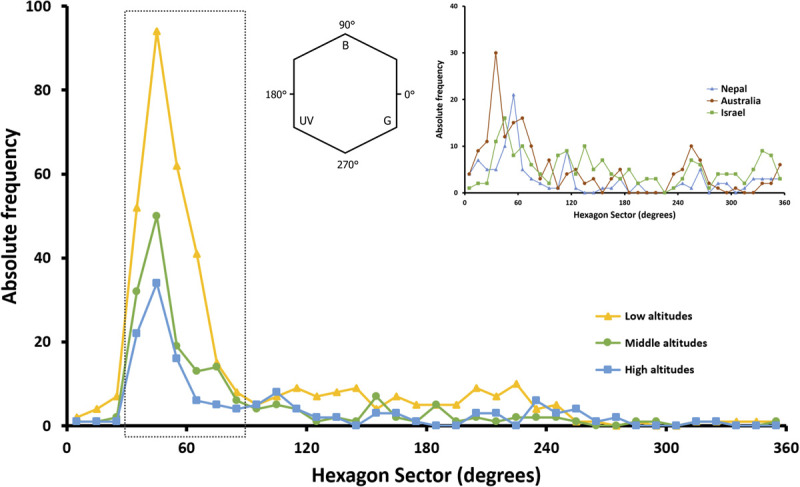
Absolute frequency distribution of floral color loci in 10° sector of bee color hexagon as proposed by Chittka et al. (1994). Distributions for species from low, middle and high altitudes in Taiwan (yellow, green and blue symbols and lines) are plotted, and compared to the global floral color distribution in hexagon color space (inset). The inset figure shows the distribution of species across the Australian continent (brown symbols and line; data from Dyer et al., 2012; Shrestha et al., 2019b), along sub-tropical and sub-alpine transects of the Nepalese Himalayas (blue symbols and line; data from Shrestha et al., 2014) and Israel (green symbols and line; data from Chittka et al., 1994). The dotted rectangle encloses the bee-blue and bee-blue–green sectors of the color space (30°–90°, see inset) where honeybees (Giurfa et al., 1995), bumblebees (Raine and Chittka, 2005) and the stingless bee *Tetragonula carbonaria* (Dyer et al., 2016) all demonstrate their strongest innate color preference.

The most predominant peak of absolute frequency for floral color loci of all Taiwanese floras (i.e., from low to high altitudes) in bee hexagon sector occurred around 60°, which lies on the bee’s dominant preference regions (Figure 7). In addition, when compared to the global floral color distribution, Taiwanese data exhibited no other distinct peaks outside the bee preference regions.

### Floral Color Assembly (FCA)

In order to test if floral colors in Taiwan show evidence of either over-dispersion or clustering, we compared the actual mean pairwise distance (MPD_color_) of the color loci in bee hexagon to the nulls MPD_color_ based on random assembly. We found the actual MPD_color_ of high-altitude species (0.245) was larger than that (0.227) of the mean of nulls MPD_color_ (random assembly), and its *p*-value was 0.930 (Table 1). This suggested that the floral colors of high-altitude species may have diverged from each other, i.e., floral color over-dispersion. On the other hand, floral colors of middle-altitude species are significantly clustered (*p* = 0.040), whereas MPD_color_ of low-altitude species appeared to be best explained by a random assembly (*p* = 0.599) (Table 1).

**TABLE 1:** Summary of floral color assembly (FCA) for three altitudes zones in Taiwan considering bee visit species and other insect visit species in high-altitude.

			**Mean pairwise distance (MPD_color_)**		
		***N***	**Actual**	**Nulls (mean ± sd)**	**P**
Among altitudes	Low	399	0.228	0.227 ± 0.005	0.599
	Middle	186	0.209	0.227 ± 0.010	0.040
	High	142	0.245	0.227 ± 0.012	0.930
High-altitude species	Bee-visit	41	0.260	0.227 ± 0.024	0.950
	Insect-visit	66	0.267	0.227 ± 0.019	0.980

**P*-value is determined by the rank of the actual MPD_color_ in 1000 nulls MPD_color._ Euclidean distance of each pairwise color loci in bee hexagon were the distance analyzed here.*

Given that not all plant species in high-altitude were pollinated by bees as other insect pollinators like flies are relatively more frequent in cooler environments (Totland, 1993; Arnold et al., 2009b; Lord et al., 2013; Paudel et al., 2016; Shrestha et al., 2016), we specifically evaluated the FCA of the species visited by either bees or other insects (see Supplementary Method S5). If the calculated MPD_color_ and relative *p*-value increase, this indicates that high-altitude plant species associating with the bees or other insects exhibit more divergent floral colors. Indeed, we found that the MPD_color_ and relative *p*-values of both bee visit (MPD_color_ = 0.260, *p* = 0.950) and insect visit (MPD_colo__*r*_ = 0.267, *p* = 0.980) species in high-altitude has increased (Table 1), showing that floral colors exhibited stronger over-dispersed within high-altitude species visited by bee and insect group.

### Phylogenetic Structure

To clarify if relatedness of species accounted for the floral color over-dispersion within high-altitude plant community, we analyzed the comparative phylogenetic structure of species in the low, mid and high-altitude zones by calculating the mean pairwise distance (MPD_phylo_) and mean nearest taxon distance (MNTD_phylo_) (details in Table 2). Both actual MPD_phylo_ and MNTD_phylo_ for species in low-altitude were close to the mean of nulls MPD_phylo_ and MNTD_phylo_ (indicative of random assembly), and the *p*-values (0.639 and 0.645) also suggested that the phylogenetic structure of low-altitude species approached to random assembly (Table 2). The MPD_phylo_ for middle-altitude species was close to the mean of nulls MPD_phylo_ (*p* = 0.540), whereas the MNTD_phylo_ was much lower than the mean of nulls MNTD_phylo_ (*p* = 0.098) (Table 2). These revealed that middle-altitude species were randomly assembled across the entire phylogenetic tree, but clustered toward the tips of the phylogenetic tree. Further, we found the MPD_phylo_ value of high-altitude species (260.6) was lower than the mean of nulls MPD_phylo_ (267.0) (*p* = 0.174), this indicated that high-altitude species are more closely related to each other across the entire phylogenetic tree (Table 2). Additionally, actual MNTD_phylo_ of high-altitude species was significantly lower than that of the mean of nulls MNTD_phylo_ (*p* = 0.004) (Table 2), which supported the evidence that high-altitude species were significantly clustered toward the tips of the phylogenetic tree.

**TABLE 2:** Summary of the phylogenetic structure of low, middle and high-altitude species in Taiwan.

**MPD_phylo_**				
**Altitude**	***N***	**Actual**	**Nulls (mean ± sd)**	**P**

Low	399	268.1	267.0 ± 3.0	0.639
Middle	186	267.8	267.1 ± 5.7	0.54
High	142	260.6	267.0 ± 6.8	0.174
**MNTD_phylo_**				
Low	399	94	93.3 ± 2.1	0.645
Middle	186	106.4	111.8 ± 4.2	0.098
High	142	105.3	118.9 ± 5.0	0.004

*The *P*-value is determined by the rank of the actual MPD_*phylo*_ (or MNTD_*phylo*_) in 1000 nulls MPD_*phylo*_ (or MNTD_*phylo*_). Larger *P*-value indicates stronger phylogenetic over-dispersion, while lower *P*-value indicates stronger phylogenetic cluster. Phylogenetic distance calculated from the phylogenetic tree was the distance analyzed here.*

### Phylogenetic Signal

The *K* values for two color descriptors (color hue, color contrast) that are known to be key factors in flower signaling were significantly deviated from random expectation (*K* = 0) and lower than that expected by Brownian motion evolution (*K* = 1) at low and high-altitude (Table 3). As all *K* values of low and high-altitudes (0.275∼0.454) were closer to zero than one, this suggested that the key descriptor values were more divergent to each other among closely related species in low and high-altitudes (i.e., weak phylogenetic signal). Moreover, Mantel test of correlation supported this weak phylogenetic signal at high altitude (*r* = 0.008, *p* = 0.417). This suggested that floral colors are liable, thus these closely related species do not necessarily have similar floral colors and may adapt to optimal pollinator in a particular environment. Interestingly, *K* value for color hue (*K* = 0.629) of middle altitude species did not deviate from 1 (Table 3), indicating floral colors of closely related species in middle altitude are more similar as that expected by Brownian motion evolution. Consistently, the Mantel test detected a relatively stronger correlation between phylogenetic distance and color distance of the middle altitude species (*r* = 0.125, *p* = 0.005). These data thus revealed a stronger phylogenetic signal, i.e., closely related species in middle-altitude plant community shared more similar floral colors than that in a low and high-altitude plant community.

**TABLE 3:** Phylogenetic signal for the floral color descriptors of species in low, mid, and high-altitude of Taiwan.

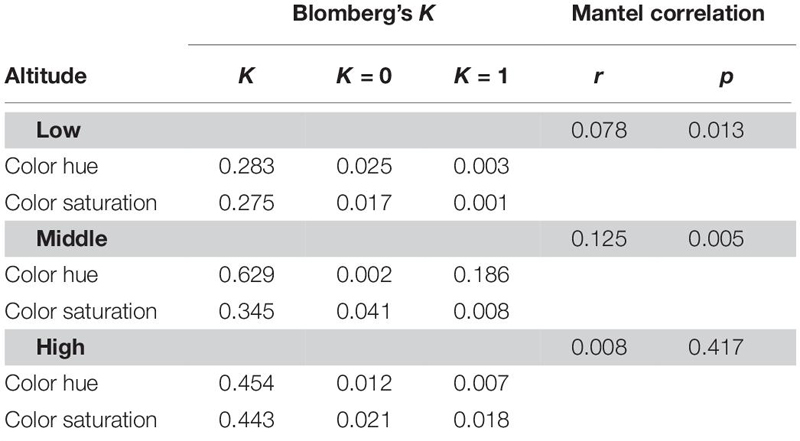

*For each color descriptor, we calculated Blomberg’s *K* value and tested whether the *K* value significantly deviated from zero (*K* = 0, no phylogenetic signal) or from one (*K* = 1, indicative of Brownian motion evolution) (significance level *P* < 0.05). Color hue and color saturation are floral color descriptors derived from color loci in bee hexagon.*

## Discussion

How flowers evolved color signals in different environments is important for understanding the plant–pollinator interactions that have occurred in the past and driven evolutionary processes (Fenster et al., 2004), and what might happen in the future with changing climatic conditions and/or pollinator distributions (Hegland et al., 2009; Shrestha et al., 2018). Based on large scales studies, species in tropical regions have been proposed to exhibit more diverse colors compared to the higher latitudes, and this may result from greater strength of biotic interaction in tropics to accelerate the evolution of coloration (Schemske et al., 2009; Dalrymple et al., 2015). However, previous island studies away from the tropics tend to show low FCD.

In the current study, we considered the uniquely placed island of Taiwan and used a hexagon color space model to plot flower loci and subsequently calculate FCD as the minimum convex polygon (MCP) encapsulating all loci. Our data revealed that the overall FCD of TW was 0.558, which is greater than AUS (0.498), but less than JPN (0.588) or NPL (0.783). Thus, despite the species richness of a mountainous tropical island with close proximity to mainland plant sources that we hypothesized should lead to high color diversity within Taiwan, it appears the data falls in the middle of FCD values for our comparative country data sets. We found that ca. 33% of the floras in our collected TW samples are endemic species, which have MCP (0.400) (Supplementary Table S4) and accounted for 72% of the overall FCD in TW, suggesting that flower color signaling is very consistent to our comparative data sets from different parts of the world. We also observed that within TW there was evidence of changes in color signaling with increasing altitude as considering low (0–900 m a.s.l.), middle (1500–2200 m a.s.l.) and high-altitude regions (2800–3300 m a.s.l.) the respective FCD values were 0.546 (399 species), 0.270 (186 species) and 0.304 (142 species) (Figure 6). Such a change did not appear to be caused simply by the number of species since at high-altitude there was a higher FCD, but actually a lower number of plant species compared to the mid-altitude region.

Taiwan is an atypical island as its low isolation level allows plant species to readily migrate to TW from subtropical Asia (e.g., China) and tropical Asia (e.g., Philippines) (Hsieh, 2002; Huang, 2011), and there is ample habitat diversity to host a range of potential pollinators. The co-shared floras covered 95% of the total FCD in TW (Supplementary Table S4). Our results suggest that observed differences in FCD at different altitudes may be mediated by lineage diversity, rather than plant species richness. The evidence for this is threefold. Firstly, we found FCD in low altitudes was greater than that in middle and high altitudes. This was because some particular color floras belonging to the families, e.g., Aristolochiaceae, Zingiberaceae, Fabaceae and Rubiaceae (*Mussaenda*) (Figure 3), were common in low altitudes but less frequent or absent in middle and high altitudes, and the color loci the flowers of these plants were often distributed toward the outer regions of hexagon color space resulting in an increase the FCD at low altitudes (Figure 6). Secondly, the phylogenetic structure of low-altitude floras was of over-dispersed, whilst the phylogenetic structure of high-altitude floras showed evidence of being clustered (Table 2). This implies that lineages of low-altitude floras contain more diverse groups than that of high-altitude floras do. Thirdly, the results of our permutation test revealed that the greater FCD in low altitude was neither due to the larger sample size, nor species richness (Supplementary Figure S2). When considering lineage diversity as the key explanation for why FCD changed with altitude, our finding is consistent with the findings of previous studies in Norway and Nepal as the lineage diversity could be represented by the amount of family and genera. The greater FCD in low altitudes of Norway was linked to greater lineage diversity (24 families, 47 genera) than in high altitudes (13 families, 14 genera) (Bergamo et al., 2018) (Supplementary Table S5). Similarly, as lineages are more diverse in flowering samples from high altitude (25 families, 45 genera) than that from low altitude (17 families, 38 genera) of Nepal, greater FCD was instead observed in high altitude (Shrestha et al., 2014) (Supplementary Table S5). Taken together these comparative findings suggest that lineage diversity is a key factor mediating FCD.

Lázaro et al. (2008) reported that flowers of white and yellow appearance to a human observer are represented by a more diverse range of plant species when considering the total number of plant species in an environment. Color trapping experiments in alpine Australia (Pickering and Stock, 2003) found that white and yellow flowers were the top two choices for multiple pollinators. Interestingly we also found that human white (bee BLUE-GREEN) and yellow (bee UV-GREEN) flowers were more dominant in Taiwan (Figure 5A). White and yellow flowers are also dominant on other islands like the temperate island of Japan (Makino and Yokoyama, 2015), some islands and mountains of southern Hemisphere (Webb and Kelly, 1993; Bernardello et al., 2001; Bischoff et al., 2013b), and these colors are also the most frequent flower hues on larger continental studies (Dyer, 1996; Arnold et al., 2009a; Dyer et al., 2012; Shrestha et al., 2014). An exception case is the study of MI (Shrestha et al., 2016) where no bees have ever been present, and the flowers of MI show a distinctive long wavelength yellowish color that fits with known long wavelength color preferences of flies; the only animal pollination vector on MI. Similarly, orchids on mainland Australia that are pollinated by flies show a distinctive long-wavelength reflection, whilst bee-pollinated orchids from the same community environment display color signals close to the spectral profiles of bee pollinated flowers around the world (Shrestha et al., 2019a). Thus, color preferences of insect pollinators may be a driving factor of flower color signaling. Such spectra fit with measured color preferences in major bee genera like *Apis* sp. and *Bombus* sp., as well as phylogenetically separated bee species like the stingless bee *Tetragonula carbonaria* (Figure 7). Interestingly, such color preferences have been observed to be biologically relevant considering the foraging decisions of naïve bees visiting real flowers (Dyer et al., 2019), providing a plausible explanation for how insect preferences do influence plant fitness and flower color signaling (Giurfa et al., 1995; Shrestha et al., 2020). In addition, floral color loci in the three altitudinal regions of TW we tested consistently and predominantly fall in the sector of bee color space preferred by bees (Figure 7). The increased FCD at higher compared to middle altitudes in TW suggests that the increased frequencies of alternative pollinators like flies may promote some diversification of flower color signaling, and future work could seek to test plant–pollinator networks to understand how signaling may be influenced by different insects with communities (Kantsa et al., 2017; Shrestha et al., 2019a,b).

Floral color over-dispersion has been reported for some alpine plant communities around the world (McEwen and Vamosi, 2010; Shrestha et al., 2014). Our result was in agreement with McEwen and Vamosi (2010) who found four of five alpine plant communities in Canada exhibited a trend towards floral color over-dispersion. Similarly, Shrestha et al. (2014) reported floral colors were significantly over-dispersed in high altitudes, whilst tending to be clustered at low altitudes in Nepal. Our results are also consistent with previous studies which reported the bees have driven the floral color over-dispersion within alpine plant communities in Nepal (Shrestha et al., 2014) and contribute to the floral color diversification of sympatric *Pedicularis* sp. in Hengduan Mt. (China) (Eaton et al., 2012).

Based on our phylogenetic-informed analysis, we found high altitude floras more closely related to each other than expected by the random assembly (i.e., phylogenetic cluster) (Table 2). However, floral color of these related species actually exhibited higher divergence than that predicted by neutral Brownian motion evolution (i.e., weak phylogenetic signal) (Table 3). Consistently, a community-based study in Nepal (Shrestha et al., 2014), and a clade-based study in Andean Solanaceae (Muchhala et al., 2014) also reported that floral color over-dispersion was mainly attributed to the divergent floral colors among sympatric closely related species.

Previous studies suggest that floral color over-dispersion most typically occurs through either character displacement or competitive exclusion (Emerson and Gillespie, 2008; Sargent and Ackerly, 2008), although it is often difficult to distinguish these two ecological processes at community level. In the current study if floral color over-dispersion had resulted from competitive exclusion, we will expect that closely related species are not likely to co-exist as they would compete with each other, thus the phylogenetic structure of the community should tend to be over-dispersed (Cavender-Bares et al., 2004; Sargent and Ackerly, 2008). On the other hand, if character displacement occurred, closely related species should evolve divergent floral colors perhaps due to reduce competition for pollinators, therefore a weak phylogenetic signal is expected (Revell et al., 2008; Muchhala et al., 2014). We found that the high altitudes floras tend to be phylogenetically clustered rather than over-dispersed (Table 2), and their floral colors exhibited weak phylogenetic signal (Table 3) which is consistent with character displacement that facilitated the co-existence of similar species. In addition, our comparative results suggest that bees appear to be the major pollinator in Taiwan consistent with other large studies around the world (Supplementary Figure S1) where the most frequently observed flower colors fit with the innate preferences of bees (Figure 7). Thus results from a large tropical–subtropical island reveal flower color evolution around the world appears to have most frequently followed a similar trajectory.

## Data Availability Statement

The datasets presented in this study can be found in online repositories. Dryad Dig. Repos. https://doi.org/10.5061/dryad.63xsj3v08.

## Author Contributions

E-CY, C-NW, MS, and AD developed the concept. K-CT, E-CY, and C-NW collected the data. MS and AD contributed data from Australia and Nepal. K-CT, MS, AD, and C-NW did data analysis and drafted the final version of the manuscript. All authors contributed to review the manuscript.

## Conflict of Interest

The authors declare that the research was conducted in the absence of any commercial or financial relationships that could be construed as a potential conflict of interest.

## Supplementary Material

Supplementary Figure 1 Marker point calculation.

Supplementary Figure 2 Frequency distribution of the areas of MCP in permutation test.

Supplementary Table 1 Detailed information of the sampling sites.

Supplementary Table 2 Details of the list of the 727 flowering plant species in our current study, including the altitude and location is provided is “.csv” file.

Supplementary Table 3 Studies used to provide subfamilial topology in the phylogenetic tree.

Supplementary Table 4 Summary of the percentage for species in our sample co-sharing with other geographic region.

Supplementary Table 5 FCD for Nepal, Norway, Japan, and Australia.

Supplementary Method 1 Details of floral color measurement.

Supplementary Method 2 Details of MPD_color_ and MNTD_phylo_ for phylogenetic structure.

Supplementary Method 3 Details of the Blomberg’s *K* and Mantel test.

Supplementary Method 4 Procedure of permutation test.

Supplementary Method 5 Identification of flower visitors of high-altitude species.
