# Erratum: Floral Color Diversity: How Are Signals Shaped by Elevational Gradient on the Tropical–Subtropical Mountainous Island of Taiwan?

**DOI:** 10.3389/fpls.2021.662738

**Published:** 2021-02-18

**Authors:** Frontiers Production Office

**Affiliations:** Frontiers Media SA, Lausanne, Switzerland

**Keywords:** flowers, bee vision, phylogeny, community, altitude, tropical–subtropical, island

Due to a production error, there was a mistake in Table 2 as published. The heading mistakenly contained “B =” which should have been removed. The corrected heading and Table 2 appear below.

**Table 2:** Summary of the phylogenetic structure of low, middle and high-altitude species in Taiwan.

**MPD_**phylo**_**						
	**Altitude**	***N***	**Actual**	**Nulls (mean ±sd)**	***P***
	Low	399	268.1	267.0 ± 3.0	0.639
	Middle	186	267.8	267.1 ± 5.7	0.54
	High	142	260.6	267.0 ± 6.8	0.174
**MNTD**_**phylo**_						
	Low	399	94	93.3 ± 2.1	0.645
	Middle	186	106.4	111.8 ± 4.2	0.098
	High	142	105.3	118.9 ± 5.0	0.004

*The P-value is determined by the rank of the actual MPD_phylo_ (or MNTD_phylo_) in 1000 nulls MPD_phylo_ (or MNTD_phylo_). Larger P-value indicates stronger phylogenetic over-dispersion, while lower P-value indicates stronger phylogenetic cluster. Phylogenetic distance calculated from the phylogenetic tree was the distance analyzed here*.

The publisher apologizes for this mistake. The original article has been updated.

