# Supplementary material for: Floral Color Diversity: How Are Signals Shaped by Elevational Gradient on the Tropical–Subtropical Mountainous Island of Taiwan?
Source: Front Plant Sci. 2020 Dec 17;11:582784. doi: 10.3389/fpls.2020.582784 (PMC7773721; doi:10.3389/fpls.2020.582784)
Supplement: Supplementary Figure 1 — Marker point calculation. [file Table_1.docx]

**Supporting information**

**Floral colour diversity: how are signals shaped by elevational gradient on the tropical-subtropical mountainous island of Taiwan?**

King-Chun Tai^1^, Mani Shrestha^2*^, Adrian G Dyer^2,3^, En-Cheng Yang^4^, Chun-Neng Wang^1,5*^

^1^Department of Life Science, National Taiwan University, Taipei, 10087, Taiwan (R.O.C.)

^2^School of Media and Communication, RMIT University, Melbourne, VIC., Australia

^3^Department of Physiology, Monash University, Melbourne, Vic., 3800, Australia

^4^Department of Entomology, National Taiwan University, Taipei, 10087, Taiwan (R.O.C.)

^5^Instituteof Ecology and Evolutionary Biology, National Taiwan University, Taipei, 10087, Taiwan (R.O.C.)

**Author for correspondence:**  Dr. Mani Shrestha

[sh.mani@gmail.com](mailto:sh.mani@gmail.com) / [mani.shrestha@rmit.edu.au](mailto:mani.shrestha@rmit.edu.au)

Professor Chun-Neng Wang

Tel: +886 02 33664537

Email: [leafy@ntu.edu.tw](mailto:leafy@ntu.edu.tw)

**Methods S1-S4**

**Methods S1.** Details of floral colour measurement

**Methods S2.** Details of MPD_colour_ and MNTD_phylo_ for phylogenetic structure

**Methods S3.** Details of the Blomberg’s K and mantel test

**Methods S4.** Procedure of permutation test.

**Methods S5.** Identification of flower visitors of high-altitude species.

**Table S1-S5**

**Table S1**. Detailed information of the sampling sites.

**Table S2**. Details of the list of the 727 flowering plant species in our current study, including the altitude and location is provided is “.csv” file. [Data available: *Dryad Digital Repository*,

http://doi.org/10.5061/dryad.63xsj3v08, (Tai et al., 2020)].

**Table S3:** **Studies used to provide sub−familial topology in the phylogenetic tree.**

**Table S4.** Summary of the percentage for species in our sample co-sharing with other geographic region.

**Table S5:** FCD for Nepal, Norway, Japan and Australia.

**Figure S1:** Marker point calculation.

**Figure S2:** Frequency distribution of the areas of MCP in permutation test.

**Appendix** A: Data available [*Dryad Digital Repository*,

http://doi.org/10.5061/dryad.63xsj3v08, (Tai et al., 2020)].

**References S1**

**-------------------------------------------**

**Methods S1:** *Details of floral colour measurement*

We used a black holder (Ocean Optics Inc., RPH-1, USA) to fix the probe at a 45° angle. Spectrophotometer was calibrated with a UV-reflecting BaSO_4_ (Ocean Optics Inc., USA) as white standard and black paperboard as dark standard.

We collected 8 to 10 fresh flowers of each species and then measured the reflectance spectrum of the larger displaying part, which was the petal in general but the sepal, bract or spathe in some species. If the petals were smaller than the diameter of the probe, many petals were arranged like fish-scale on the adhesive tape to achieve the minimum area for measurement by following the method used in (Arnold et al., 2010). Additionally, we also measured the reflectance spectra of the leaves for all samples in our study, which were averaged and used a green foliage background in calculation of colour loci in bee hexagon.

**Method S2:** *Details of* MPD_colour_ and *MNTD_phylo_ for phylogenetic structure*

If the actual MPD_colour_ value is larger than the mean of nulls MPD_colour_, this infers floral colours tend to be divergent with each other (floral colour over-dispersion). On the contrary, if the actual MPD_colour_ value is lower than the mean of nulls MPD_colour_, the floral colours tend to be similar to each other (floral colour cluster). The higher the p-value indicates stronger floral colour over-dispersion, while a lower p-value indicates stronger floral colour cluster (McEwen and Vamosi, 2010; Kluge and Kessler, 2011).

Because MPD_phylo_ is used to evaluate the pattern of phylogenetic structure across the entire phylogeny, we additionally calculated the mean nearest taxon distance (MNTD_phylo_) of species in low, middle and high altitudes. MNTD_phylo_ is the branch length between one species and its closest relative on phylogeny, so it is more sensitive to the pattern that species cluster towards the tips of the phylogenetic tree. If actual MNTD_phylo_ value is smaller than mean of nulls MNTD_phylo_, it infers that species tend to cluster towards the tips of the phylogenetic tree, but may or may not cluster across the entire tree. Its p-value is determined by the rank of the actual MNTD_phylo_ in 1000 nulls MNTD_phylo_, therefore, larger p-value indicates stronger phylogenetic over-dispersion, while lower p-value indicates stronger phylogenetic cluster. All calculation of the MNTD_phylo_ and its significance p-value is computed with function ‘ses.mntd’ in R package PICANTE (Kembel et al., 2010).

**Method S3:** *Details of the Blomberg’s K and mantel test*

We examined phylogenetic signal with the commonly used Blomberg’s K in previous studies (Blomberg et al., 2003; Muchhala et al., 2014; Shrestha et al., 2014). This enables us to examine whether patterns of flower colour difference can be attributed to their phylogenetic relatedness. We calculated the Blomberg’s K values of the two key floral colour descriptors: colour hue (Shrestha et al., 2014), and colour contrast (referred to as saturation in some studies) in bee hexagon (Lunau, 1990; Kantsa et al., 2017) of low, mid and high-altitude floras in our study using the function ‘phylosig’ in R package PHYTOOLS (Revell, 2012). We also performed Mantel test to evaluate the phylogenetic signal (Muchhala et al., 2014; Shrestha et al., 2014; Bergamo et al., 2018), which aims to examine the correlation between the phylogenetic distances and the colour distances (in bee hexagon) of all pairwise species. Mantel test is conducted with function ‘mantel.rtest’ in R package ADE4.

To interpret the Blomberg’s K values, if K value is close to zero, it indicates that the similarity of trait values within related species tends to show no difference to that within random selected species, i.e. this trait tends to lack phylogenetic signal. Increasing K value indicates that a trait has greater phylogenetic signal. When K=1, trait values within related species are similar with each other as that predicted by Brownian motion evolution. If K>1, trait values within related species are more similar than that expected under Brownian motion evolution, i.e. this trait is highly constrained by phylogenetic relatedness and exhibits strong phylogenetic signal.

To evaluate the significance of K values, firstly, we test whether each K value is deviated from 0 by comparing it with the null distribution of the K values from random permutation of the tip labels of phylogeny. Secondly, to test if the K value is deviate from 1, we compare it to the K values from simulation of the traits under Brownian process, following the method established by (Revell et al., 2007).

For mantel test, the significance of correlation coefficient is determined by 1,000 times permutation along the metric Dray and Dufour (2007). Larger correlation coefficient and significant p-value indicate the stronger correlation between phylogenetic distance and colour distance, i.e. related species (small phylogenetic distance) display similar floral colours (small colour distance) which is equivalent to stronger phylogenetic signal.

**Methods S4*:*** *Procedure of permutation test.*

We used permutation test to solve the argument that unequal sample size between different altitudes (from low to high: n=399, 186 vs 142) may bias the areas of MCP. In this test, we will randomly resample 142 colour loci from the pool comprising all loci of floras from low-altitude for 10,000 times, and then calculate the area of MCP of each resample (nulls MCP). After that, the actual area of MCP for high-altitude floras (0.304) will be compared to the distribution of nulls MCP for low-altitude floras. If the actual MCP of high-altitude floras (0.304) is smaller than 95% of the nulls MCP, it suggests that the area of MCP for high-altitude floras persist to be lower than that of low-altitude floras, irrespective to the sample size. Otherwise, if actual area of MCP for high-altitude floras is not smaller than 95% of the nulls MCP, this indicates that the smaller area of MCP for high-altitude floras can probably be attributed to the smaller sample size. Because middle-altitude floras have larger sample size and lower area of MCP (n=186, MCP=0.270) than high-altitude floras (n=142, MCP=0.304), a permutation test for this data is not required. We conducted the permutation test with R version 3.4.4.

**Methods S5:** *Identification of flower visitors of high-altitude species.*

Observation of flower visitors of high-altitude species were done from April till September in 2017 (Wang *et al.*, unpublished data). We set up 19 square quadrats (each one was 2m×10m) at our high-altitude sampling site (Hohuan Mt.), and these quadrats have covered the major vegetation types in high-altitudes of Taiwan. In total, we observed 5908 individual insects visiting 93 different plant species. As not all of the plant species in quadrats are native species, we identified 66 native species visited by insects (all kinds of insects) and regarded them as ‘insect-visit’ species. Likewise, we identified 41 native species visited by hymenopteran insects and regarded them as ‘bee-visit’ species. In addition, not all 142 high-altitude species in our study (with spectra data) were surrounded in the quadrats, some of the quadrants thus lacked any flower visitor data.

**Table S1.** Detailed information of the sampling sites.

| **Sites** | **Range of altitude** | **Locality** | **Number of species** |
| --- | --- | --- | --- |
| **Low-altitude (0-1200 m)** | | | |
| Yangmingshan National Park | 250~887 m | 25.1901N, 121.5198E | 70 |
| Northeast Coast National Scenic Area | 0~336 m | 25.0171N, 121.9427E | 6 |
| Taipei Botanical Garden (TPBG) | 0 m | 25.0322N, 121.5096E | 73 |
| Shizaitoushan | 678~857 m | 24.9034N, 121.4935E | 22 |
| Wulai (Xinxian and Yunxian Park) | 230~500 m | 24.8445N, 121.5428E | 4 |
| Manyueyuan National Forest Recreation Area | 300 m | 24.8304N, 121.4446E | 3 |
| Fushan Research Centre (FSBG) | 600 m | 24.7605N, 121.5831E | 28 |
| Taroko National Park | 54~370 m | 24.1789N, 121.5489E | 18 |
| Kenting National Park | 0~227 m | 21.9605N, 120.8108E | 74 |
| Green Island | 0~228 m | 22.6645N, 121.4891E | 32 |
| Orchid Island | 0~480 m | 22.0212N, 121.5724E | 69 |
| **Middle-altitude (1200-2400 m)** | | | |
| Lidongshan | 1528~1914 m | 24.6929N, 121.3051E | 10 |
| Taipingshan National Forest Recreation Area | 1954~2000 m | 24.4954N, 121.5336E | 34 |
| Dasyueshan National Forest Recreation Area | 1580~2218 m | 24.2394N, 120.9715E | 22 |
| Meifeng | 1743~2100 m | 24.0911N, 121.1764E | 35 |
| Sunlinksea Forest Recreation Area | 1560~1690 m | 23.6337N, 120.7959E | 54 |
| Alishan National Forest Recreation Area | 1640~2201 m | 23.5184N, 120.8068E | 31 |
| **High-altitude (2400-3600 m)** |  |  |  |
| Hohuan Mt. | 2848~3275 m | 24.1616N, 121.2871E | 142 |

**Table S2.** Details of the list of the 727 flowering plant species in our current study, including the altitude and location is provided is “.csv” file [Data deposited on Dryad database, doi: http://doi.org/10.5061/dryad.63xsj3v08, (Tai et al., 2020)].

**Table S3:** Studies used to provide sub−familial topology in the phylogenetic tree.

**Taxon Reference(s)**

Acanthaceae MacDade & Moody (1999)

Amaryllideae Meerow & Snijman (2001)

Apocynaceae Sennblad & Bremer (2002)

Asteraceae Kim *et al.* (2005), Panero & Funk (2008)

Gnaphalieae Bayer *et al.* (2002)

Bignoniaceae Olmsetead *et al.* (2009), Olmstead (2012)

Campanulaceae

*Wahlenbergia* Prebble *et al.* (2011)

Ericales Anderberg *et al.* (2002)

Fabaceae Sulman *et al.* (2003), Lavin *et al.* (2005)

*Acacia* Murphy *et al.* (2010)

Goodeniaceae Jabaily *et al.* (2012)

Iridaceae Reeves *et al.* (2001)

Lamiaceae Bendiksby *et al*. (2011)

*Clerodendrum* Steane *et al*. (2004)

Lecythidaceae Morton *et al*. (1997)

Malvaceae Alverson *et al.* (1999)

Marantaceae Prince & Kress (2006)

Myrtaceae Biffin *et al.* (2010)

Orchidaceae Cameron *et al*. (1999), Cameron (2004), Gustaffson *et al*. (2010)

Pittosporaceae Chandler *et al.* (2007)

Rubiaceae Bremer & Eriksson (2009)

Solanaceae Olmstead *et al*. (2008)

Verbenaceae Marx *et al*. (2010)

Zingiberaceae

*Alpinia* Kress *et al*. (2005)

**Table S4:** Summary of the percentage for species in our sample co-sharing with other geographic regions, and endemic to Taiwan. Data was extracted from the Flora of Taiwan, second edition (Flora of Taiwan Second Edition eds., 1993).

| **Biogeography/Country** | **Taiwan** |  |  | **Altitude (m)** |  |
| --- | --- | --- | --- | --- | --- |
|  |  |  | **Low** | **Middle** | **High** |
| Tropical Asia | 23.20% |  | 33.60% | 11.83% | 9.15% |
| FCD (MCP) | 0.481 |  | 0.481 | 0.069 | 0.055 |
| Subtropical Asia (Subtropical China) | 29.60% |  | 29.30% | 38.71% | 18.31% |
| FCD (MCP) | 0.373 |  | 0.333 | 0.200 | 0.158 |
| Temperate Asia (Temperate China and Japan) | 9.60% |  | 8.00% | 10.22% | 13.38% |
| FCD (MCP) | 0.315 |  | 0.164 | 0.042 | 0.213 |
| Endemic Species | 32.60% |  | 22.60% | 34.95% | 57.75% |
| FCD (MCP) | 0.400 |  | 0.352 | 0.204 | 0.253 |
| Cosmopolitan Species | 5.00% |  | 6.52% | 4.30% | 1.41% |
| FCD (MCP) | 0.108 |  | 0.060 | 0.054 | 0 |

Note: MCP of the species co-sharing with Tropical Asia and Subtropical Asia (total 52.8%) was 0.528.

**Table S 5:** FCD for Nepal, Norway, Japan and Australia, which was represented by the area of MCP. Number in bracket indicates the number of plant species in each altitudinal category.

| **Altitude\Country** | **Nepal** | **Norway** | **Japan** | **Australia** |
| --- | --- | --- | --- | --- |
| Low | 0.394 (46) | 0.402 (43) | — | — |
| mid | — | 0.313 (22) | — | — |
| high | 0.747 (59) | 0.083 (10) | — | — |
| All | 0.783 (105) | 0.433 (77) | 0.588 (212) | 0.498 (201) |


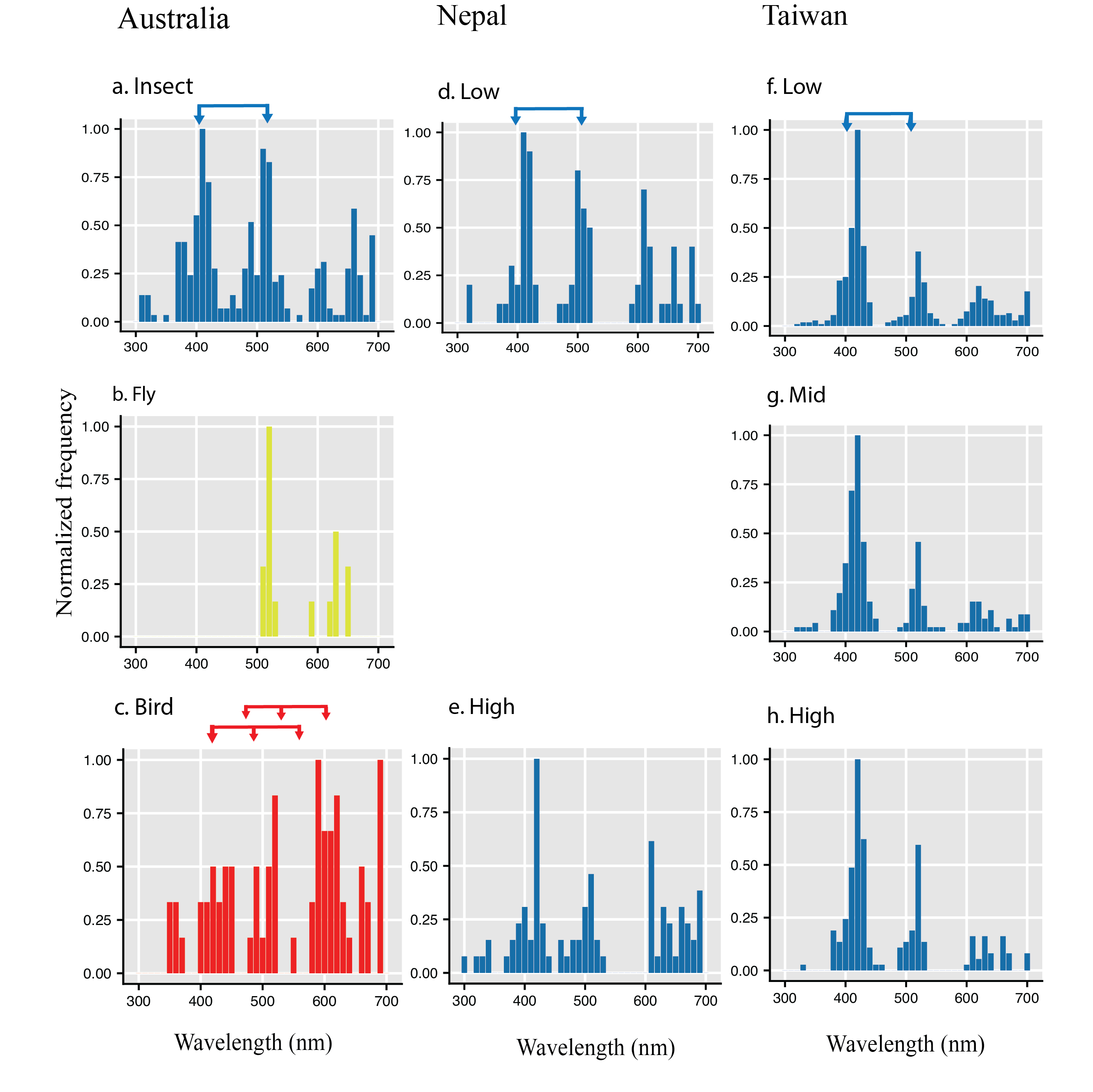


**Figure S1:** Marker point calculation and evidence that bees are the primary pollinator of flowering plants at low, middle and high altitudes in Taiwan. It is known that animal pollination is the primary driver of flower coloration, although in a complex natural environment it may be difficult to ascertain which properties of animal colour vision most influence flower evolution because different animals have different colour visual systems (Kemp et al., 2015). The most likely pollinators of flowers in Taiwan include bees, flower visiting flies and/or birds Bees have a phylogenetically ancient trichromatic color visual system based on UV, Blue and Green photoreceptors which is characterized by optimal processing near photoreceptor overlap at about 400 and 500nm (von Helversen, 1972; Peitsch et al., 1992; Chittka and Menzel, 1992; Briscoe and Chittka, 2001; Dyer et al., 2012). Panel (a) shows bee discrimination optimal processing by the two joined blue arrows which closely match flower spectral signature Marker Points for insect pollinated flowers from Australia (data from Shrestha et al. 2013). Flower visiting flies have tetrachromatic vision and a strong innate preference for long wavelength reflecting colors (Lunau, 2014), and subsequently fly pollinated flowers (b) lack having a 400nm MP (data from Shrestha et al., 2016). Birds have tetrachromatic colour vision (Hart and Hunt 2007) and bird pollinated flowers (c) subsequently have different discrimination that promotes flowers typically reflecting long wavelength red colour characterized by marker points most frequent at 600nm (data from Shrestha et al., 2013). In the Nepalese Himalayas at both low (d) and high latitude (e) flower spectral signals are best characterised by bee colour vision (data from Shrestha et al. 2014). In Taiwan considering Low (f), Mid (g) and High (h) altitudes we also observe flower color signals most typically show characteristic spectral signal MPs at wavelengths consistent with pollination by bees. Other potential pollinators like butterflies have higher dimension colour vision that to date has shown no evidence of fitting the spectral profiles of flowers (Dyer et al. 2012).


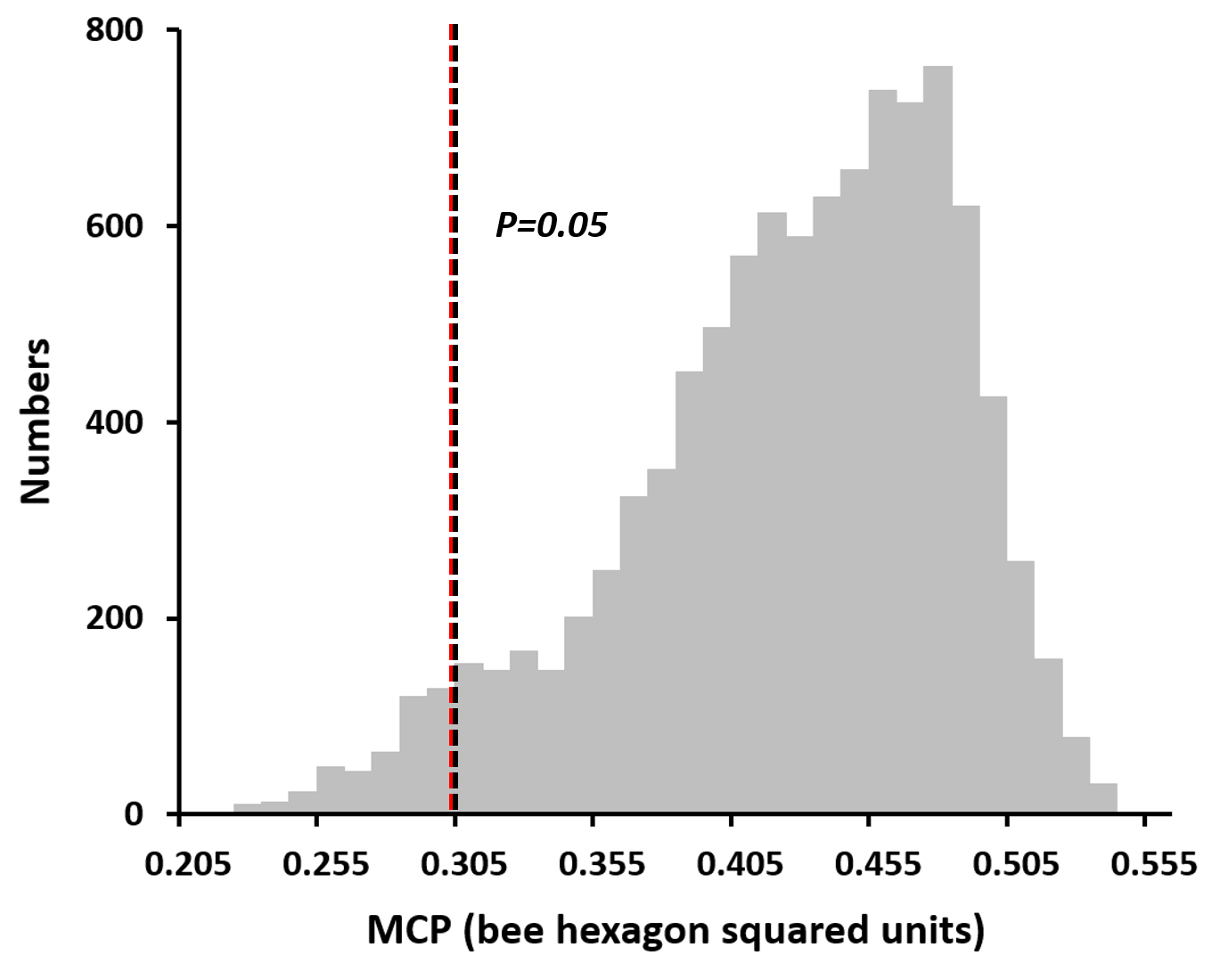


**Figure S2:** Frequency distribution of the areas of MCP in permutation test (10,000 times). Black dot line indicated the threshold (MCP=0.305) for the lower 5% nulls area of MCP. Red dot line indicated the actual area of MCP for high-altitude floras (MCP=0.304, P<0.05). The actual area of MCP for high-altitude floras was lower than 95% of the nulls area of MCP for low-altitude floras, this suggested that the area of MCP of high-altitude floras persisted to be lower than that of low-altitude, irrespective to sample sizes.

**Reference S1**

Alverson WS, Whitlock BA, Nyffeler R, Bayer C, Baum DA. 1999. Phylogeny of the core Malvales: evidence from ndhF sequence data. Am. J. Bot. 86: 1474-86

Anderberg AA, Rydin C, Källersjö M. 2002. Phylogenetic relationships in the order Ericales s.l.: analyses of molecular data from five genes from the plastid and mitochondrial genomes. Am. J. Bot. 89: 677-87

Arnold, S. E. J., Faruq, S., Savolainen, V., Mcowan, P. W., and Chittka, L. (2010). Fred: The floral reflectance database — a web portal for analyses of flower colour (floral reflectance database). PLoS ONE 5, e14287. doi: 10.1371/journal.pone.0014287

Barker NP, Weston PH, Rutschmann F, Sauquet H. 2007. Molecular dating of the ‘Gondwanan’ plant family Proteaceae is only partially congruent with the timing of the break-up of Gondwana. J. Biogeogr. 34: 2012-27

Bayer RJ, Greber DG, Bagnall NH. 2002. Phylogeny of Australian Gnaphalieae (Asteraceae) based on chloroplast and nuclear sequences, the trnL intron, trnL/trnF intergenic spacer, matK, and ETS. Syst. Bot.: 801-14

Bendiksby M, Thorbek L, Scheen A-C, Lindqvist C, Ryding O. 2011. An updated phylogeny and classification of Lamiaceae subfamily Lamioideae. Taxon 60: 471-84

Biffin E, Lucas EJ, Craven LA, Ribeiro da Costa I, Harrington MG, Crisp MD. 2010. Evolution of exceptional species richness among lineages of fleshy-fruited Myrtaceae. Ann. Bot. 106: 79-93

Birgitta Bremer, Torsten Eriksson. 2009. Time tree of Rubiaceae: Phylogeny and dating the family, subfamilies, and tribes. Int. J. Plant Sci. 170: 766-93

Cameron KM, Chase MW, Whitten WM, Kores PJ, Jarrell DC, et al. 1999. A phylogenetic analysis of the Orchidaceae: evidence from rbcL nucleotide sequences. Am. J. Bot. 86: 208-24

Cameron KM. 2004. Utility of plastid psaB gene sequences for investigating intrafamilial relationships within Orchidaceae. Mol. Phylogen. Evol. 31: 1157-80

Chandler GT, Plunkett GM, Pinney SM, Cayzer LW, Gemmill CEC. 2007. Molecular and morphological agreement in Pittosporaceae: phylogenetic analysis with nuclear ITS and plastid trnL–trnF sequence data. Aust. Syst. Bot. 20: 390-401

Clements MA, Tupac Otero J, Miller JT. 2011. Phylogenetic relationships in Pterostylidinae (Cranichideae: Orchidaceae): combined evidence from nuclear ribomsomal and plastid DNA sequences. Aust. J. Bot. 59: 99-117

Dray, S., and Dufour, A.-B. (2007). The ade4 package: implementing the duality diagram for ecologists. Journal of statistical software 22, 1-20. doi: 10.18637/jss.v022.i04

Flora of Taiwan Second Edition Eds. (1993). Flora of Taiwan, Second Edition. Taipei: Editorial Committee of the Flora of Taiwan, Second Edition.

Gustafsson A, Verola C, Antonelli A. 2010. Reassessing the temporal evolution of orchids with new fossils and a Bayesian relaxed clock, with implications for the diversification of the rare South American genus Hoffmannseggella (Orchidaceae: Epidendroideae). BMC Evol. Biol. 10: 1-13

Hopper SD, Brown AP. 2004. Robert Brown's Caladenia revisited, including a revision of its sister genera Cyanicula, Ericksonella and Pheladenia (Caladeniinae: Orchidaceae). Aust. Syst. Bot. 17: 171-240

Jabaily RS, Shepherd KA, Gustafsson MHG, Sage LW, Krauss SL, et al. 2012. Systematics of the Austral-pacific family Goodeniaceae: Establishing a taxonomic and evolutionary framework. Taxon 61: 419-36

Janes JK, Steane DA, Vaillancourt RE, Duretto MF. 2010. A molecular phylogeny of the subtribe Pterostylidinae (Orchidaceae): resolving the taxonomic confusion. Aust. Syst. Bot. 23: 248-59

Jones D. 2006. A complete guide to native orchids of Australia including the Island Territories. Reed New Holland publication, Sydney, Australia.

Kembel, S. W., Cowan, P. D., Helmus, M. R., Cornwell, W. K., Morlon, H., Ackerly, D. D., Blomberg, S. P., and Webb, C. O. (2010). Picante: R tools for integrating phylogenies and ecology. Bioinformatics 26, 1463-1464. doi: 10.1093/bioinformatics/btq166

Kim K-J, Choi K-S, Jansen RK. 2005. Two chloroplast DNA inversions originated simultaneously during the early evolution of the sunflower family (Asteraceae). Mol. Biol. Evol. 22: 1783-92

Kluge, J., and Kessler, M. (2011). Phylogenetic diversity, trait diversity and niches: species assembly of ferns along a tropical elevational gradient. Journal of Biogeography 38, 394-405. doi: 10.1111/j.1365-2699.2010.02433.x

Kress WJ, Liu A-Z, Newman M, Li Q-J. 2005. The molecular phylogeny of Alpinia (Zingiberaceae): a complex and polyphyletic genus of gingers. Am. J. Bot. 92: 167-78

Lavin M, Herendeen PS, Wojciechowski MF. 2005. Evolutionary Rates Analysis of Leguminosae Implicates a Rapid Diversification of Lineages during the Tertiary. Syst. Biol. 54: 575-94

Marx HE, O’Leary N, Yuan Y-W, Lu-Irving P, Tank DC, et al. 2010. A molecular phylogeny and classification of Verbenaceae. Am. J. Bot. 97: 1647-63

Mast AR, Milton EF, Jones EH, Barker RM, Barker WR, Weston PH. 2012. Time-calibrated phylogeny of the woody Australian genus Hakea (Proteaceae) supports multiple origins of insect-pollination among bird-pollinated ancestors. Am. J. Bot. 99: 472-87

McDade LA, Moody ML. 1999. Phylogenetic relationships among Acanthaceae: evidence from noncoding trnL-trnF chloroplast DNA sequences. Am. J. Bot. 86: 70-80

Meerow AW, Snijman DA. 2001. Phylogeny of Amaryllidaceae tribe Amaryllideae based on nrDNA ITS sequences and morphology. Am. J. Bot. 88: 2321-30

Morton C, Mori S, Prance G, Karol K, Chase M. 1997. Phylogenetic relationships of Lecythidaceae: a cladistic analysis using rbcL sequence and morphological data. Am. J. Bot. 84: 530

Murphy DJ, Brown GK, Miller JT, Ladiges PY. 2010. Molecular phylogeny of Acacia mill. (Mimosoideae: Leguminosae): Evidence for major clades and informal classification. Taxon 59: 7-19

Olmstead RG, Bohs L, Migid HA, Santiago-Valentin E, Garcia VF, Collier SM. 2008. A molecular phylogeny of the Solanaceae. Taxon 57: 1159-81

Olmstead RG, Zjhra ML, Lohmann LG, Grose SO, Eckert AJ. 2009. A molecular phylogeny and classification of Bignoniaceae. Am. J. Bot. 96: 1731-43

Olmstead RG. 2013. Phylogeny and biogeography in Solanaceae, Verbenaceae and Bignoniaceae: a comparison of continental and intercontinental diversification patterns. Bot. J. Linn. Soc. 171: 80-102

Panero JL, Funk VA. 2008. The value of sampling anomalous taxa in phylogenetic studies: Major clades of the Asteraceae revealed. Mol. Phylogen. Evol. 47: 757-82

Prebble JM, Cupido CN, Meudt HM, Garnock-Jones PJ. 2011. First phylogenetic and biogeographical study of the southern bluebells (Wahlenbergia, Campanulaceae). Mol. Phylogen. Evol. 59: 636-48

Prince LM, Kress WJ. 2006. Phylogenetic relationships and classification in Marantaceae: insights from plastid DNA sequence data. Taxon 55: 281-96

Reeves G, Chase MW, Goldblatt P, Rudall P, Fay MF, et al. 2001. Molecular systematics of Iridaceae: evidence from four plastid DNA regions. Am. J. Bot. 88: 2074-87

Revell, L. J., Johnson, M. A., Schulte, J. A., Kolbe, J. J., and Losos, J. B. (2007). A phylogenetic test for adaptive convergence in rock-dwelling lizards. Evolution 61, 2898-2912. doi: 10.1111/j.1558-5646.2007.00225.x

Sennblad B, Bremer B. 2002. Classification of Apocynaceae s.l. According to a new approach combining Linnaean and phylogenetic taxonomy. Syst. Biol. 51: 389-409

Steane DA, de Kok RPJ, Olmstead RG. 2004. Phylogenetic relationships between Clerodendrum (Lamiaceae) and other Ajugoid genera inferred from nuclear and chloroplast DNA sequence data. Mol. Phylogen. Evol. 32: 39-45

Sulaiman SF, Culham A, Harborne JB. 2003. Molecular phylogeny of Fabaceae based on rbcL sequence data: with special emphasis on the tribe Mimoseae (Mimosoideae). Asia Pacific Journal of Molecular Biology and Biotechnology 11: 9-35

Tai, K.-C., Shrestha, M., Dyer, A. G., Yang, E.-C., and Wang, C.-N. Data from: Floral colour diversity in the tropical-subtropical mountainous island of Taiwan. Dryad Digital Repository. (2020) https://doi.org/10.5061/dryad.63xsj3v08

van den Berg C, Goldman DH, Freudenstein JV, Pridgeon AM, Cameron KM, Chase MW. 2005. An overview of the phylogenetic relationships within Epidendroideae inferred from multiple DNA regions and recircumscription of Epidendreae and Arethuseae (Orchidaceae). Am. J. Bot. 92: 613-24

Weston PH, Barker NP. 2006. A new suprageneric classification of the Proteaceae, with an annotated checklist of genera. Telopea 11: 314-44

Wikström N, Kainulainen K, Razafimandimbison SG, Smedmark JEE, Bremer B. 2015. A revised time tree of the asterids: Establishing a temporal framework for evolutionary studies of the coffee family (Rubiaceae). PLoS ONE 10: e0126690
